# Idiopathic Pulmonary Fibrosis Mortality Risk Prediction Based on Artificial Intelligence: The CTPF Model

**DOI:** 10.3389/fphar.2022.878764

**Published:** 2022-04-26

**Authors:** Xuening Wu, Chengsheng Yin, Xianqiu Chen, Yuan Zhang, Yiliang Su, Jingyun Shi, Dong Weng, Xing Jiang, Aihong Zhang, Wenqiang Zhang, Huiping Li

**Affiliations:** ^1^ The Academy for Engineering and Technology, Fudan University, Shanghai, China; ^2^ Department of Respiratory Medicine, Shanghai Pulmonary Hospital, Tongji University, School of Medicine, Shanghai, China; ^3^ Department of Pulmonary and Critical Care Medicine, Yijishan Hospital of Wannan Medical College, Wuhu, China; ^4^ Department of Radiology, Shanghai Pulmonary Hospital, School of Medicine, Tongji University, Shanghai, China; ^5^ Department of Medical Statistics, School of Medicine, Tongji University, Shanghai, China

**Keywords:** artificial intelligence (AI), deep learning, semantic segmentation, idiopathic pulmonary fibrosis (IPF), pulmonary fibrosis stage, disease severity grade

## Abstract

**Background:** Idiopathic pulmonary fibrosis (IPF) needs a precise prediction method for its prognosis. This study took advantage of artificial intelligence (AI) deep learning to develop a new mortality risk prediction model for IPF patients.

**Methods:** We established an artificial intelligence honeycomb segmentation system that segmented the honeycomb tissue area automatically from 102 manually labeled (by radiologists) cases of IPF patients’ CT images. The percentage of honeycomb in the lung was calculated as the CT fibrosis score (CTS). The severity of the patients was evaluated by pulmonary function and physiological feature (PF) parameters (including FVC%pred, DLco%pred, SpO2%, age, and gender). Another 206 IPF cases were randomly divided into a training set (*n* = 165) and a verification set (*n* = 41) to calculate the fibrosis percentage in each case by the AI system mentioned previously. Then, using a competing risk (Fine–Gray) proportional hazards model, a risk score model was created according to the training set’s patient data and used the validation data set to validate this model.

**Result:** The final risk prediction model (CTPF) was established, and it included the CT stages and the PF (pulmonary function and physiological features) grades. The CT stages were defined into three stages: stage I (CTS≤5), stage II (5 < CTS<25), and stage III (≥25). The PF grades were classified into mild (a, 0–3 points), moderate (b, 4–6 points), and severe (c, 7–10 points). The AUC index and Briers scores at 1, 2, and 3 years in the training set were as follows: 74.3 [63.2,85.4], 8.6 [2.4,14.8]; 78 [70.2,85.9], 16.0 [10.1,22.0]; and 72.8 [58.3,87.3], 18.2 [11.9,24.6]. The results of the validation sets were similar and suggested that high-risk patients had significantly higher mortality rates.

**Conclusion:** This CTPF model with AI technology can predict mortality risk in IPF precisely.

## Introduction

The survival of IPF patients varies considerably. Some are stable for a long time, some progress slowly, and some exacerbate acutely, leading to short-term death (Ley et al., 2011; Raghu et al., 2018). A widely accepted method of assessing disease severity and estimating prognosis remains absent (Gonnella et al., 1984).

Currently, severity assessment models of IPF mainly include the following: 1)The CRP (clinical-radiographic-physiologic) scoring model proposed by Leslie C. Watters et al. (Watters et al., 1986; Watters et al., 1987) in 1986, which consists of seven variables: the degree of dyspnea, X-ray chest radiograph quantitative score, forced vital capacity (FVC), forced expiratory volume in one second (FEV_1_), intrathoracic gas volume (Vtg), diffusing capacity of the lung for carbon monoxide (DLco) and lung volume (VA) ratio (DLco/VA), and the alveolar–arterial oxygen partial pressure difference (AaPO2) in the resting state. The CRP model includes numerous parameters, the calculation is complex, and it is difficult to identify fibrotic lesions from chest X-ray images. King et al. (2001) improved the CRP scoring system in 2001, by adding parameters such as gender, age, smoking status, and clubbing, which further increased the complexity of the evaluation. 2)In 2002, Wells et al. (2003) proposed CPI (composite physiologic index), which only used the lung function parameters to assess the severity of interstitial lung disease (ILD); however, its calculation formula is complicated, and its clinical application is limited. 3) Ley et al. (2012) proposed a GAP (gender, age, and physiologic variables) model based on gender, age, FVC, and DLco. However, the essential CT data was still not included. 4) Okuda et al. (2013) proposed to use arterial partial pressure of oxygen (PaO2) and oxyhemoglobin saturation (SaO2%), two leading arterial blood gas indicators to assess severity; however, CT, lung function, and other essential parameters are still missing from this approach. Hence, it is necessary to establish a precise and easy-performing model to evaluate and predict the prognosis of IPF.

In recent years, artificial intelligence (AI), especially deep learning, has been evolving rapidly and has achieved remarkable results in computer vision (CV). Traditional computer-based CT analysis provided objective quantitation of IPF disease programs such as CALIPER. Jacob et al. (2017) used it to measure disease severity with feature engineering, which usually involves subjective experience and might lead to non-optimal results. Compared with the traditional CV method, the deep learning-based method learns the features by itself with an end-to-end architecture that avoids human subjective feature selection, and this usually archives the state-of-the-art results (O’Mahony et al., 2019). One of the essential tasks of CV is semantic segmentation, which can be thought of as pixel-wise classification. Deep learning-based semantic segmentation has been widely applied in biomedical image processing (Ronneberger et al., 2015) in areas such as the lung (Hofmanninger et al., 2020; Handa et al., 2021), kidney (Bazgir et al., 2020), brain tumor (Myronenko and Hatamizadeh, 2020), sublingual vein (Xiong et al., 2020), and prostate (Yoo et al., 2019) and achieved state-of-the-art results. We extended semantic segmentation into pulmonary fibrosis image analysis by training a deep learning model that segments fibrosis tissue regions in chest CT images automatically and calculates the fibrosis tissue percentage of the entire lung (patent application no. 202010985175.8). Combining the aforementioned pulmonary function and physiological feature (PF) parameters , which have been proved to have a good prognostic value and are easy to be accessed clinically, we set up a new comprehensive framework for evaluating the severity of pulmonary fibrosis (patent no: ZL 2019 1 0514972.5). We conducted clinical verification (ChiCTR-RRC-17010683), which achieved accurate pulmonary fibrosis severity assessment and prognosis evaluation (software registration no. 6406807).

## Methods

### Study Cohorts

**FIGURE 1:**
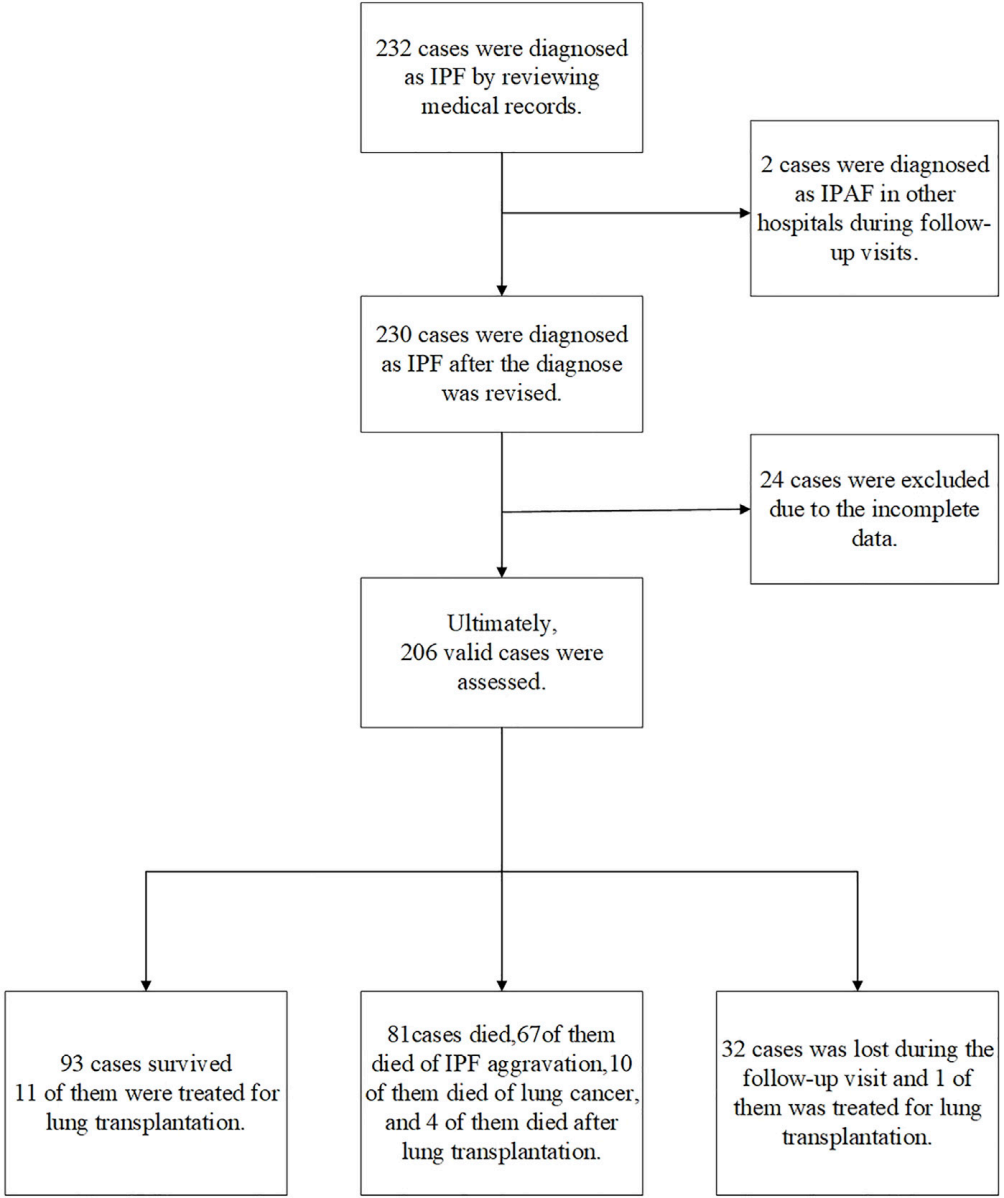
Case screening process. In total, 232 cases were diagnosed as IPF according to the 2018 IPF diagnosis and treatment guidelines. A total of 26 patients were excluded, two patients were diagnosed as interstitial pneumonia with autoimmune features (IPAF) during follow-up; 24 patients had incomplete CT and lung function data. Finally, 206 cases were included in the retrospective analysis (including 16 cases of lung transplantation): 93 surviving cases, including 11 lung transplants; 81 deaths, out of which 10 died from lung cancer, 67 died from acute exacerbation of IPF, and 4 died after lung transplantation; and 32 patients failed to follow up, including one failed to follow up after lung transplantation.

For testing the AI system that we established (patent application no. 202010985175.8), we did a retrospective analysis of 232 patients diagnosed with IPF from 1 January 2011 to 31 January 2020 in the Department of Respiratory Medicine, Shanghai Pulmonary Hospital. IPF diagnosis of these cases was confirmed by the criteria of the 2018 IPF International Guidelines (Raghu et al., 2018). Data of gender, age, lung function, fingertip SpO2% (or SaO2% measured by arterial blood gas analysis), chest CT, occupation, and smoking history were recorded. All patients were followed up in outpatient clinics or *via* phone, including the patient’s survival status, time of death (the year and month), cause of death, whether there were other complications, whether undergoing lung transplantation, and the time of lung transplantation. The deadline for follow-up was 1 August 2020. After follow-up, finally, 206 qualified cases were involved in the study. The patient screening process and follow-up are shown in Figure 1. This study was approved by the Institutional Ethics Committee of Shanghai Pulmonary Hospital (No. K17-016).

### Development of the Mortality Risk Prediction Model for IPF

Based on the IPF diagnosis guidelines in 2018 (Raghu et al., 2018), the honeycomb lung extent and scope of the disease presented on the CT images of IPF patients are essential indicators for predicting IPF mortality (Flaherty et al., 2003; Best et al., 2008; Raghu et al., 2011; Rosas et al., 2011). The thickness of HRCT sections was 1–2 mm; section spacing was 2 cm. Patients were in the supine position. The minimum exposure was 200 mA per second. First, we established a deep learning AI model by a neural network (Ronneberger et al., 2015) (lung segmentation network, LSN) to calculate the proportion of honeycomb in the total lung. The LSN was trained by identifying the 102 IPF patients’ honeycomb lesion area labeled by radiologists manually (Supplementary Material S1). Then, we used this AI model to quantify the extent of honeycomb lung lesions for another 206 patients. The CT images were also reviewed separately by two radiologists, who were blinded to the clinical information and the deep learning model results. Both radiologists were board-certified diagnostic radiologists, who were majoring in chest radiology. The observers evaluated the extent of the honeycomb and gave the manual-CT score results.

The patient’s PF parameters are indispensable for prognosis estimation (Wells et al., 2003; Ley et al., 2012). After analysis of the pros and cons of existing scoring systems (CRP, GAP, CPI, and JRS) shown in Table 1, we chose five parameters, namely, FVC%pred, DLco%pred, SpO2%, age, and gender, to evaluate the severity of the patient’s disease (patent no: ZL 2019 1 0514972.5). These parameters have a significant predictive value and can be accessed easily in clinical practice. According to previous studies (Watters et al., 1986; Watters et al., 1987; King et al., 2001; Wells et al., 2003; Ley et al., 2012; Okuda et al., 2013), we formed a multi-parameter severity evaluation metric (PF grading) based on PF.

**TABLE 1:** Comparison of different pulmonary staging methods.

Scoring method	Parameter														Advantage	Disadvantage
	Gender	Age	FVC%	DLco%	Tlc%	FEV_1_%	Lung capacity (vtg)	HRCT	X-ray	PaO_2_	SpO_2_%	Smoking	Clubbing finger	Extent of dyspnea		
GAP	√	√	√	√											Simple	Lack HRCT and PaO_2_ data
CPI			√	√		√									Can reflect combined emphysema	Lack HRCT and PaO_2_ data
CRP					√		√		√	√		√	√	√	Require many parameters	Complex and lack HRCT and lung function data
JRS										√	√				Simple	Lack HRCT and PaO_2_ data
Accessibility	Easy	Easy	Easy	Easy	Easy	Easy	Require a comprehensive device to measure lung function	Easy	Easy but images overlap	Require arterial blood	Easy	Difficult for quantification	Vary greatly in individuals	Require a complex scoring system and may be influenced by subjective bias		
Importance	Y	Y	Y	Y	Affected by multiple factors	Correlate to airway disease	?	Y	Y	Y	Y	?	?	Y		
Parameters in our method	√	√	√	√				√			√					

Notes: Y: the parameter is important. ? the importance of the parameter is currently unknown. √: the parameter was included in the model of this study.

SpO_2_%: oxygen saturation of peripheral blood. SpO_2_ is the resting arterial oxygen saturation measured at fingertips. FVC: forced vital capacity. FVC%pred: the percentage of the actual FVC over the predicted FVC. FEV_1_: forced expiratory volume in one second. FEV_1_%pred: the percentage of the actual FEV_1_ over the predicted FEV_1_. DLco: diffusing capacity of the lung for carbon monoxide. DLco%pred: the percentage of the actual DLco over the predicted DLco. FEV_1_/FVC%: the percentage of FEV_1_ over FVC. GAP (gender, age, and physiologic variables) stage followed the recommendation by Brett Ley, and a higher stage represented a greater death risk. CPI: composite physiologic index. In 2002, Athol U. Wells and others proposed to use CPI, which combined chest CT and pulmonary functional parameters, to assess the severity of interstitial lung diseases (ILDs). A higher CPI represents a more severe ILD. CRP: clinical-radiographic-physiologic. Leslie C. Watters et al. published the CRP system in 1986. JRS: Ryo Okuda et al. proposed the IPF staging method in 2004. HRCT: high-resolution computed tomography.

### Statistical Method

The Spearman correlation coefficient was used to analyze the correlation between CTS and lung function parameters, namely, FVC%pred, DLco%pred, SpO2%, and CPI. The patient’s survival time was calculated from the evaluation time to the endpoint event, which was death due to lung disease or lung transplantation, measured in months. According to survival time, X-tile software (internal cross-validation method) was used to find the optimal CT score threshold to classify patients in three CT stages.

Lung transplantation was considered the most effective treatment for IPF (Thabut et al., 2009), so lung transplantation was considered a competing risk event and used the competing risk (Fine–Gray) model for establishment and evaluation of the disease prognosis prediction model as follows: 1) based on the total number of our cases, the modeling parameters were selected, by referring to the existed literature (Roecker, 1991; Zhang et al., 2018); all 206 cases were randomly divided into the training set (165 cases) and the verification set (41 cases). 2) CT staging, PF staging, and CTPF comprehensive staging were used as predictors. We compared the accuracy of the model with GAP staging proposed by Brett Ley and MD and established four mortality risk prediction models based on Fine–Gray regression analysis for training set data, namely the CT staging model, PF grading model, CTPF staging model, and GAP staging model. The predictive accuracy of the risk model was assessed by calculating the area under ROC curve (AUC) and Brier score. 3) The validation set was used to validate the four models. 4) A nomogram was drawn referred to some reports (Zhang et al., 2017; Zhang et al., 2018) to show the 1, 2, and 3-year survival rates of the CTPF model for patients visually with different CT stages and PF grades.

The statistical software used in this study was IBM SPSS24.0, Stata/MP14.0 X-Tile, and R3.4.3.

## Results

### Patient Baseline Clinical Characteristics

Following the process shown in Figure 1, we screened 232 cases of IPF patients. Among them, 206 cases met the scoring requirements and were included in the CTPF staging verification: 93 cases survived, 81 cases died, 32 cases failed to follow up, and 16 cases received lung transplantation. Table 2 shows the primary characteristics of the patients. The average age is 64.1 ± 7.9 (years), and the average survival time is 28.7 ± 19.3 (months). Most patients are male (196/206, 95.1%), and most of them have a history of smoking (156/206, 75.7%). The average CT score is 14.1 ± 11.30 (ranges from 0.04 to 52.3).

**TABLE 2:** Patients’ general clinical characteristics.

Patient data (n-206)	Value
Median age years	64.1 ± 7.9
Male/female	196/10
Smokers/non-smokers	150/56
Survival time (months)	28.7 ± 19.3
SpO_2_%	95.2 ± 3.5
FVC%pred	71.9 ± 20.1
FEV_1_%pred	75.1 ± 20.6
DLco%pred	52.0 ± 28.4
FEV_1_/FVC%	83.5 ± 7.8
CT score values by AI	14.1 ± 11.30
CT score values by radiologists	24.5 ± 13.8
CT stage I/II/III	56/114/36
PF stage a/b/c	95/80/31
GAP stage I/II/III	108/63/35
CPI	44.6 ± 21.0

Notes: Measurement data are presented as mean ± standard deviation (SD). Count data are presented as percentage or proportion.

SpO_2_%: oxygen saturation of peripheral blood. SpO_2_ is the resting arterial oxygen saturation measured at fingertips. FVC: forced vital capacity. FVC%pred: the percentage of the actual FVC over the predicted FVC. FEV_1_: forced expiratory volume in one second. FEV_1_%pred: the percentage of the actual FEV_1_ over the predicted FEV_1_. DLco: diffusing capacity of the lung for carbon monoxide. DLco%pred: the percentage of the actual DLco over the predicted DLco. FEV_1_/FVC%: the percentage of FEV_1_ over FVC. CT score values were calculated by AI according to the method in the article. CT-based stage: the stage was determined by using CT score values by AI following the criteria described in Table 3. PF-based grade: the grade was determined by using the pulmonary function and physiological parameters (age, gender, FVC%pred, DLco%pred, and SpO2%) and following the description in Table 3. The grade was defined as (a) mild, (b) moderate, and (c) severe. GAP (gender, age, and physiologic variables) stage followed the recommendation by Brett Ley, and a higher stage represented a greater death risk. CPI: composite physiologic index. In 2002, Athol U. Wells and others proposed to use CPI, which combined chest CT and pulmonary functional parameters, to assess the severity of interstitial lung diseases (ILDs). A higher CPI represents a more severe ILD.

### Test the CT Score Calculated by AI

**FIGURE 2:**
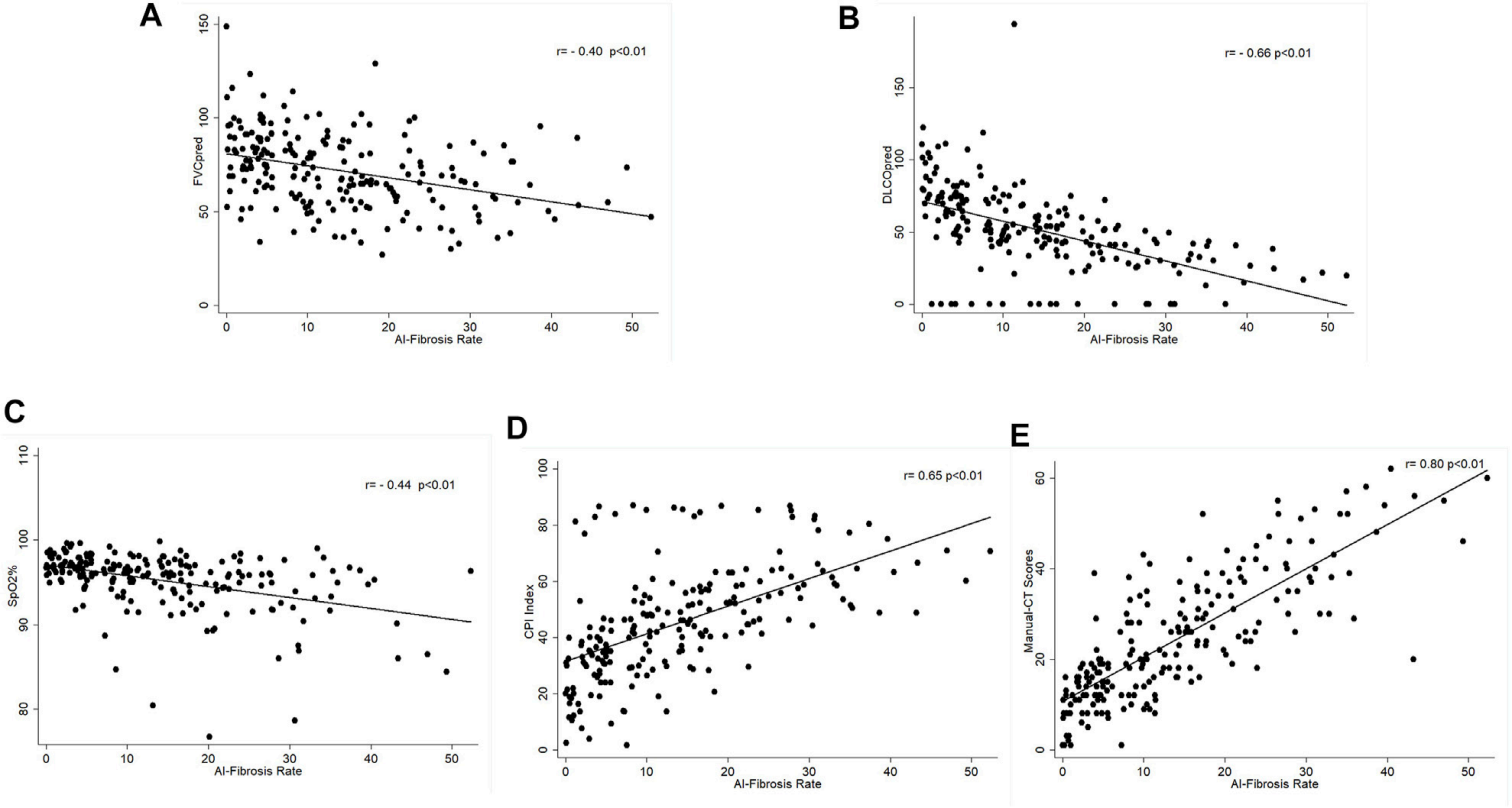
Correlation of AI–CT fibrosis score and lung function parameters. **(A)** Correlation between CT-score and FVC%pred, Spearman correlation coefficient rs = -0.40, *p* < 0.01; **(B)** correlation between CT-score and DLco%pred, Spearman correlation coefficient rs = -0.66, *p* < 0.01; **(C)** correlation between CT-score and SpO2%, Spearman correlation coefficient rs = -0.44, *p* < 0.01; **(D)** correlation between CT-score and CPI, Spearman correlation coefficient rs = 0.65, *p* < 0.01; and **(E)** correlation between CT-score and manual-CT scores by radiologists, Spearman correlation coefficient rs = 0.80, *p* < 0.01.

The fibrosis segmentation network (FSN) was the essential component of deep learning, which performed the semantic segmentation of fibrosis regions in the CT images, and is the basis of further calculation, such as CT scores (CTS) and FSN’s performance, shown in Supplemental Material 2. Figures 2A–D show that the CTS was negatively correlated to FVC%pred (rs = -0.40, *p* < 0.01), DLco%pred (rs = -0.66, *p* < 0.01), and SpO2% (rs = -0.44, *p* < 0.01) and positively correlated with the existing CPI (rs = 0.65, *p* < 0.01) which reflects the severity of the patient’s disease. In addition, the CTS was closely related to manual-CT scores by radiologists and Spearman correlation coefficient rs = 0.80, *p* < 0.01 (Figure 2E.). It indicates that the CT scoring system designed in this study properly reflects the severity of pulmonary fibrosis.

### Establishment of the CTPF Model

According to the survival time of all patients, we used X-tile software to find the cut-off points of CT scores and are calculated as 5.6 and 25.4, which divide the patients into three groups, and the survival rates of the three groups are statistically different (χ2 = 27.985, *p* < 0.05). To facilitate clinical application, we tried to take integer cut-off points, i.e., 5 and 25. We used the two cut-off point CT scores (5.6, 25.4) and (5,25) to establish the prediction model and found that both scores have the same prediction efficiency. For ease of clinical use, we chose the latter. So, the three groups were as follows: stage I (CTS<5), stage II (5 < CTS<25), and stage III (CTS>25).

After analyzing the pros and cons of existing scoring systems (CRP, GAP, CPI, and JRS) shown in Table 1, we chose five parameters, namely, FVC%pred, DLco%pred, SpO2%, age, and gender, to evaluate the severity of the patient’s disease and calculated the scores using PF grading to assess the severity in patients (Table 3) and prognosis.

**TABLE 3:** Criteria for CT-based pulmonary fibrosis staging and PF-based severity grading (patent no: ZL 2019 1 0514972.5).

PF Scoring criteria	SpO_2_%	FVC%pred						DLco%pred					Age (year)			Gender		Total severity score	Criteria for severity grading		
	≥95%	90–94%		≤89%		>75%	50–75%	<50%	>55%	36–55%	<36%	Cannot complete	≤60	61–65	>65	M	F	10	a (mild)	b (moderate)	c (severe)
	0	1		2		0	1	2	0	1	2	3	0	1	2	1	0		0–3	4–6	7–10
CT-based staging criteria			I		Honeycomb lesion area is <5% of the entire lung																
			II		Honeycomb lesion area is 5–25% of the entire lung																
			III		Honeycomb lesion area is >25% of the entire lung																
CTPF stage presentation example			Fibrosis stage/severity		Definition																
						II a		Fibrosis stage II and IPF severity grade a (mild)																

Notes: SpO_2_%: oxygen saturation of peripheral blood. SpO_2_% is the resting arterial oxygen saturation measured at fingertips. FVC: forced vital capacity. FVC%pred: the percentage of the actual FVC over the predicted FVC. DLco: diffusing capacity of the lung for carbon monoxide. DLco%pred: the percentage of the actual DLco over the predicted DLco.

**FIGURE 3:**
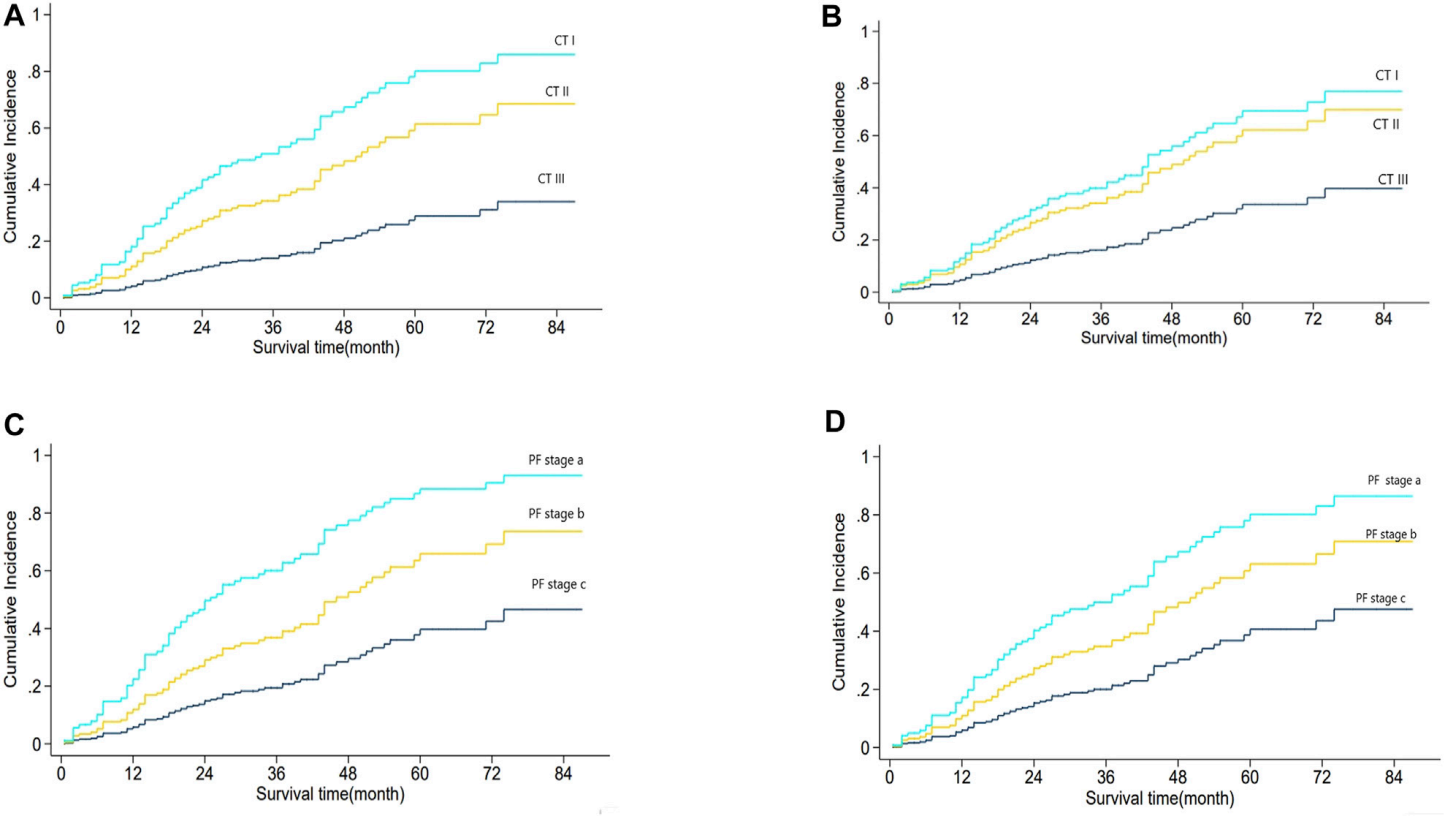
Analysis of CT stage and PF grading and mortality.(A) shows the relationship between CT staging and mortality risk based on Fine–Gray regression CT staging univariate analysis, which might be mixed with the influence of PF grade. (B) shows the same relationship in the multivariate analysis of CT staging and PF grading. Adjusted PF grading means the effect of PF grading was eliminated. The results showed that CT stage, with both PF grade adjusted and unadjusted, was positively correlated with mortality risk. (C) shows the relationship between PF grade and mortality risk based on Fine–Gray regression PF grade univariate analysis, which might be mixed with the influence of CT staging. (D) shows the same relationship between the multivariate analysis of CT staging and PF classification. CT staging adjusted means the effect of the CT stage was eliminated. The results show that the PF grade, with both CT staging adjusted and unadjusted, is positively correlated with mortality risk.

Figure 3A shows the relationship between CT staging and mortality risk in Fine–Gray univariate regression analysis, in which the effect of PF grading might be involved. Figure 3B shows the result of multi-factor analysis after eliminating the effect of PF grading, that is, the relationship between CT staging and mortality risk. In both adjusted and unadjusted cases, PF staging was positively correlated with mortality risk. Similarly, Figures 3C, D illustrate the relationship between PF grading and mortality risk in Fine–Gray regression with unadjusted and adjusted CT staging. In both cases, PF stages were positively correlated with mortality risk. We then combined the two factors to create a new mortality prediction model, the CTPF model. The score was calculated based on the five pulmonary function and physiological feature prognostic predictors, and CT scores were calculated by the AI model, which are shown in Table 3.

### Validation of the CTPF Model

Table 4 shows the patients’ clinical characteristics in the training set and validation set. There is no significant difference between the two sets. Then, classification of the training set was followed according to the CT staging, PF grading, GAP staging, and CT + PF staging. The analysis results in Table 5 show that the AUC index and Briers scores at 1, 2, and 3 years are as follows: 74.3 [63.2,85.4], 8.6 [2.4,14.8]; 78 [70.2,85.9], 16.0 [10.1,22.0]; and 72.8 [58.3,87.3], 18.2 [11.9,24.6]. The CTPF model has the best AUC index and Briers scores. The results of the validation sets were similar. The AUC index and Briers scores at 1, 2, and 3 years in the validation set are as follows: 92.0 [83.4,100.0], 8.1 [0.5,15.7]; 75.0 [57.1,92.9], 14.3 [6.6,22.1]; and 76.0 [56.8,95.2], 17.6 [9.4,25.9].

**TABLE 4:** Patients’ clinical characteristics of the training set and validation set.

Characteristic	Combined set	Training set	Validation set	*p*-value
No.[n (%)]	206 (100)	165 (80)	41 (20)	
FVCpred [mean (SD)]	71.91 (20.12)	71.95 (20.45)	71.74 (18.97)	0.953
Fibrosis rate [*median* (*Q1*,*Q3*)]	11.34 (4.61,20.74)	11.45 (4.86,20.28)	9.62 (3.92,23.2)	0.390
Emphysema rate [median (Q1,Q3)]	0.16 (0.02,1.23)	0.18 (0.02,1.23)	0.16 (0.01,1.05)	0.931
Age [median (Q1,Q3)]	64.5 (60,69)	65 (59,70)	64 (60,68)	0.542
SaO2 [median (Q1,Q3)]	96 (94.4,97.3)	96 (94.2,97.1)	96.8 (95.4,97.8)	0.156
FEV1pred [median (Q1,Q3)]	73.75 (60.7,88.7)	74.1 (60.8,87.6)	69 (60.4,91.6)	0.764
DLCO pred [median (Q1,Q3)]	51.6 (36.9,70.1)	51.5 (36.9,69.6)	51.7 (37.4,73.9)	0.977
Survivetime [median (Q1,Q3)]	27 (13,40)	26 (14,40)	27 (10,38)	0.441
GAP stage I/II/III	108/63/35	86/49/30	22/14/5	0.631
PF grade a/b/c	95/80/31	74/65/26	21/15/5	0.729
CT stage I/II/III	56/114/36	43/94/28	13/20/8	0.636

Notes: Measurement data are presented as mean ± standard deviation (SD). Count data are presented as percentage or proportion.

SpO_2_%: oxygen saturation of peripheral blood. SpO_2_ is the resting arterial oxygen saturation measured at fingertips. FVC: forced vital capacity. FVC%pred: the percentage of the actual FVC over the predicted FVC. FEV_1_: forced expiratory volume in one second. FEV_1_%pred: the percentage of the actual FEV_1_ over the predicted FEV_1_. DLco: diffusing capacity of the lung for carbon monoxide. DLco%pred: the percentage of the actual DLco over the predicted DLco. CT-based stage: the stage was determined by using the average score of the two radiologists and following the criteria described in Table 3. PF-based grade: the grade was determined by using the pulmonary function and physiological parameters (age, gender, FVC%pred, DLco%pred, and SpO2%) and following the description in Table 3. The grade was defined as (a) mild, (b) moderate, and (c) severe. GAP (gender, age, and physiologic variables) stage followed the recommendation by Brett Ley, and a higher stage represented a greater death risk.

**TABLE 5:** Discrimination of different models in the training and validation cohort.

Prediction time	Model	Training set		Validation set	
		AUC	Brier score	AUC	Brier score
1 year	CTPF	74.3 [63.2,85.4]	8.6 [2.4,14.8]	92.0 [83.4,100.0]	8.1 [0.5,15.7]
	CT	66.4 [55.4,77.4]	8.9 [2.5,15.4]	86.2 [70.9,100.0]	8.5 [0.5,16.5]
	PF	71.8 [60.7,82.9]	8.7 [2.4,15.1]	84.6 [71.6,97.5]	8.4 [0.4,16.4]
	GAP	73.5 [63.0,84.0]	8.7 [2.4,15.0]	75.3 [60.0,90.5]	8.7 [0.5,16.9]
2 years	CTPF	78 [70.2,85.9]	16.0 [10.1,22.0]	75.0 [57.1,92.9]	14.3 [6.6,22.1]
	CT	69.6 [61.6,77.5]	17.5 [11.2,23.7]	73.7 [58.0,89.4]	14.3 [7.2,21.3]
	PF	74.1 [65.4,82.7]	16.5 [10.2,22.8]	65.2 [43.8,86.6]	15.2 [6.5,23.8]
	GAP	71.2 [62.3,80.0]	17.0 [10.9,23.1]	62.3 [41.9,82.7]	15.3 [7.0,23.5]
3 years	CTPF	72.8 [58.3,87.3]	18.2 [11.9,24.6]	76.0 [56.8,95.2]	17.6 [9.4,25.9]
	CT	64.2 [51.0,77.5]	19.8 [13.2,26.5]	65.2 [46.8,83.7]	20.1 [12.1,28.1]
	PF	70.6 [56.5,84.8]	18.5 [12.0,25.0]	72.5 [54.3,90.7]	18.3 [9.7,26.9]
	GAP	69.9 [55.6,84.3]	18.7 [12.4,25.0]	68.8 [50.8,86.8]	19.0 [10.7,27.3]

Notes: CI: confidence interval. Model CT: CT-based stage was used in the univariate Fine–Gray death risk regression analysis. Model PF: PF-based grade was used in the univariate Fine–Gray death risk regression analysis. Model CTPF: CTPF comprehensive stage was used in the multivariate Fine–Gray death risk regression analysis. Model GAP: GAP stage proposed by Brett Ley was used in univariate Fine–Gray death risk regression analysis. AUC: area under curve. The AUC value reflects the model’s capability of discrimination. The higher the AUC value, the higher the model’s ability to identify the mortality risk. Brier score is an indicator that comprehensively reflects the discrimination and calibration of the model. The smaller the Brier score, the better the discrimination and calibration predicted by the model.

### Prognostic Significance of the CTPF Model

**FIGURE 4:**
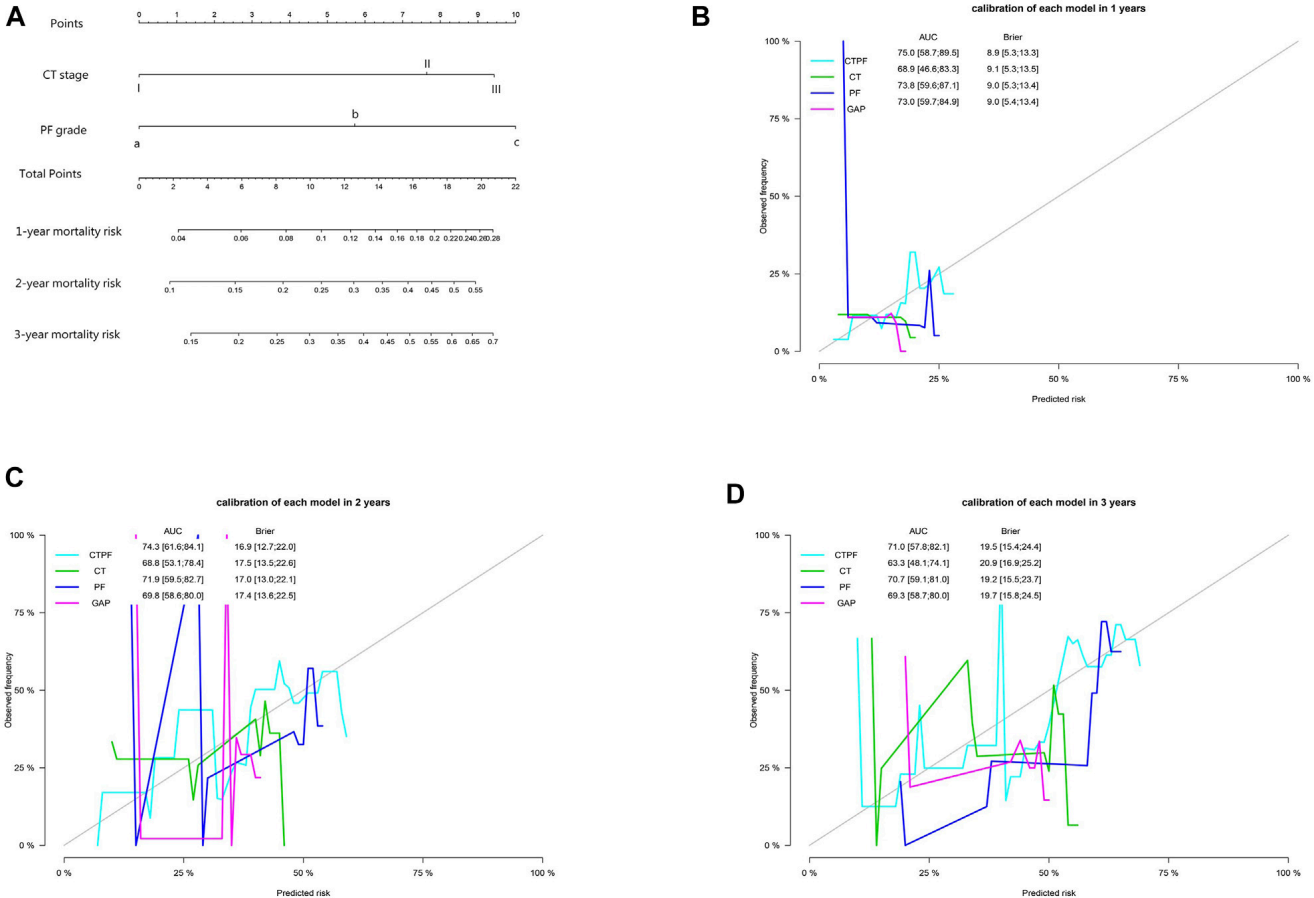
Model prediction nomogram and calibration curve. **(A)** Mortality nomogram of CTPF as the predictive model. **(B–D)** Calibration curves after cross-validation using CT staging, PF staging, CTPF comprehensive staging, and GAP staging to predict patients’ cumulative mortality risk at 1, 2, and 3 years. The CTPF prediction model has the best AUC value, Brier score, and stability.

A nomogram of death risk prediction for a CTPF prediction model and calibration curve are shown in Figure 4. The 1-year, 2-year, and 3-year cumulative survival rates of different CTPF stages based on the nomogram are shown in Table 6. The higher the PF grade, for patients with the same CT staging, the lower the cumulative survival rate and vice versa.

**TABLE 6:** CTPF model-predicted 1-, 2-, and 3-year accumulative survive rate of patients at different CTPF stage.

CTPF stage	1-year cumulative survival rate%	2-year cumulative survival rate%	3-year cumulative survival rate %
I a	96.90	91.77	89.11
I b	94.15	84.83	80.20
I c	90.68	76.56	69.88
II a	92.79	81.51	76.02
II b	86.64	67.60	59.15
II c	79.23	52.96	42.63
III a	91.23	77.84	71.46
III b	83.89	61.90	52.55
III c	75.18	45.89	35.18

Notes: CTPF, stage: CTPF-based comprehensive stage. I a: CT, stage I and PF, grade a; I b: CT, stage I and PF, grade b; I c: CT, stage I and PF, grade c; II a: CT, stage II, and PF, grade a; II b: CT, stage II, and PF, grade b; II c: CT, stage II, and PF, grade c; III a: CT, stage III, and PF, grade a; III b: CT, stage III, and PF, grade b; III c: CT, stage III, and PF, grade c.

CT I: honeycomb lesion area was <5% of the entire lung. CT II: honeycomb lesion area was 5–25% of the entire lung. CT III: honeycomb lesion area was >25%. The PF-based grade was determined by assessing the scores of age, gender, FVC%pred, DLco%pred, and SpO_2_% according to the criteria in Table 3 and adding the scores. PF (a): score 0–3. PF(b): score 4–6. PF(c): score 7–10.

**FIGURE 5:**
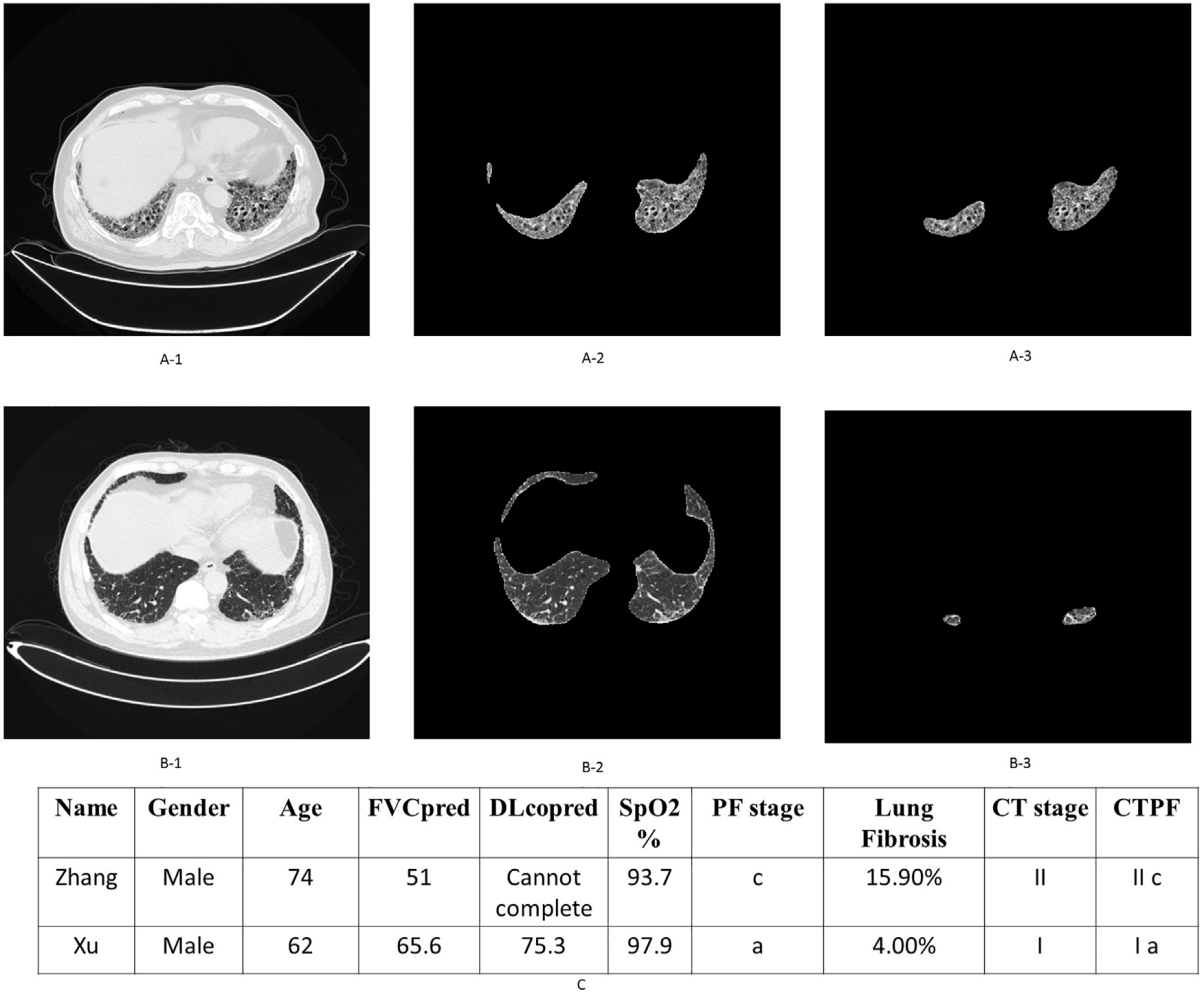
Examples of patient’s original lung CT image, honeycomb lung region segmentation, and staging. (A-1,2,3) are the original CT images, the segmented lung region, and honeycomb lung region identified by the deep learning model of patient Zhang. The corresponding stage of this patient is II c. Similarly, (B-1,2,3) are the corresponding images of patient Xu, whose stage is Ia. (C) shows their comprehensive CTPF staging; patient Zhang’s comprehensive stage is IIc; the comprehensive stage of patient Xu is Ia.

As the flow in Supplementary Figure S1 shows that all the patients’ chest CT lung images were read into the deep learning model. The model segmented the patients’ fibrotic lesion region and calculated the area percentage of the whole lung. Age, gender, FVC%pred, DLco%pred, and SpO2% data were included in the metric to calculate patient’s CTPF staging results (Figure 5), and an evaluation report (Supplementary Figure S7) was generated.

A total of two representative cases are displayed as follows: Figure 5 shows the output of the fibrosis segmentation network. Figure 5A shows a 74-year-old male patient, whose CT fibrosis score is 15.9. In Table 3, the physiological indicators of lung function (PF) correspond to seven points of severity, so his comprehensive stage is IIc; the patient died of exacerbation 23 months later. Figure 5B shows another patient, a 62-year-old male with an AI fibrosis score of 4.0. According to Table 3, the final stage of the patient is Ia. The patient is still alive after 39 months when we followed up.

As some IPF patients had also developed emphysema (Rosas et al., 2011), we trained another semantic segmentation model for pulmonary bulla and calculated its percentage of the entire lung based on the same framework with different parameters. See Supplementary Material S1 for details.

## Discussion

Current IPF staging evaluation methods shown in Table 1 are either too simple, such as GAP and JRS which cannot accurately reflect the severity of the disease and estimate prognosis because of fewer data, or too complex, such as CPR and CPI which are complex and difficult to access in clinical practice (Jacob et al., 2017). In fact, chest CT scans are one of the standard clinical examination methods in the diagnosis of IPF, and honeycomb in the lung is the most representative lesion of pulmonary fibrosis and directly related to the prognosis (Flaherty et al., 2003; Lynch et al., 2005; Best et al., 2008; Raghu et al., 2011; Rosas et al., 2011). The semi-quantitative evaluation is the most common method in practice, which requires physicians’ expertise, and is labor-intensive and time-consuming, and the results of different practitioners might vary considerably. The Cohen-weighted k values of semi-quantitative evaluation are only 0.40–0.58 (Watadani et al., 2013; Hansell et al., 2015), and both repeatability and accuracy are also low. The pulmonary fibrosis segmentation model based on deep learning in this study segmented fibrosis honeycomb accurately and automatically and calculated its percentage of the whole lung, which quantifies the essential factor of fibrosis staging. Compared with manual-CT scores evaluated by radiologists, the scores evaluated by AI were low. Due to the fact that the AI evaluation was a whole-lung range in the chest CT, the manual evaluation was usually selected for the dominant lesion section in the CT, such as the aortic arch section, tracheal bifurcation section, and lung diaphragm section.

This method has the advantages of fast incremental learning, objective and accurate quantitative calculation, efficient complete lung scanning, and high repeatability. The DSC of the model reached 77.26%, which is 8.39% higher than that of the benchmark (U-Net with the spatial pyramid pooling module) that is 68.78% (Ronneberger et al., 2015). Compared with CALIPER (Jacob et al., 2017) based on traditional CV technology, deep learning methods learn features automatically and archive better performance. Although Handa et al. (2021) adopted deep learning U-Net architecture, we enhanced it with an attention mechanism and Squeeze-Excitation Network to archive better outcomes. The running time of the CT evaluation for each patient was only 11 s, which is a significant efficiency improvement.

We selected FVC%pred, DLco%pred, SpO2%, age, and gender as five essential indicators that have been proved to have a good prognostic value and are easy to be accessed clinically to evaluate the severity of IPF. In both univariate and multivariate regression analyses, the results suggested that PF classification was an independent risk factor for predicting IPF patients’ mortality risk. The severity of each patient’s disease stage (a, b, or c) was calculated according to these five parameters. The CTPF evaluation system combines the results from CT pulmonary fibrosis staging (I, II, and III) and severity grading (a, b, and c) to form a complementary pulmonary fibrosis staging/severity grading model CTPF (Table 3). The assessment report shows the result (Figure 5.).

In the task of mortality risk prediction, the CTPF model has better AUC, Brier score, and stability than any other model (PF, CT, and GAP staging). Lung transplantation is an effective way to improve the prognosis of IPF patients (Thabut et al., 2009). However, lung transplantation itself also has a mortality risk. In 2015, Yusen et al. (2015) reported a global mortality risk of lung transplantation as 20% in 1 year and 35% in 3 years. We suggest those patients whose mortality risk (Table 6) is higher than the lung transplantation risk to consider transplantation. In this regard, our model could suggest the appropriate time window for lung transplantation.

The prognostic evaluation of pulmonary fibrosis with emphysema needs to be further analyzed in additional cases.

The major limitation of this study is lack of an external validation cohort to further evaluate the CTPF model. We are planning a multicenter clinical study in the future and hope to verify its clinical significance.

## Conclusion

The deep learning-based model calculated the percentage of fibrosis lesions of the whole lung quantitatively by segmenting the fibrosis region from chest CT images automatically, combined with the IPF severity determined by five important physiological and pulmonary function indicators. The CTPF model predicted the mortality risk for IPF patients more precisely.

## Data Availability

The raw data supporting the conclusion of this article will be made available by the authors, without undue reservation.
