# Supplementary figures and images for: Idiopathic Pulmonary Fibrosis Mortality Risk Prediction Based on Artificial Intelligence: The CTPF Model
Source: Front Pharmacol. 2022 Apr 26;13:878764. doi: 10.3389/fphar.2022.878764 (PMC9086624; doi:10.3389/fphar.2022.878764)

**Figure.S3.** Manual labeling the fibrosis lesion area with different colors


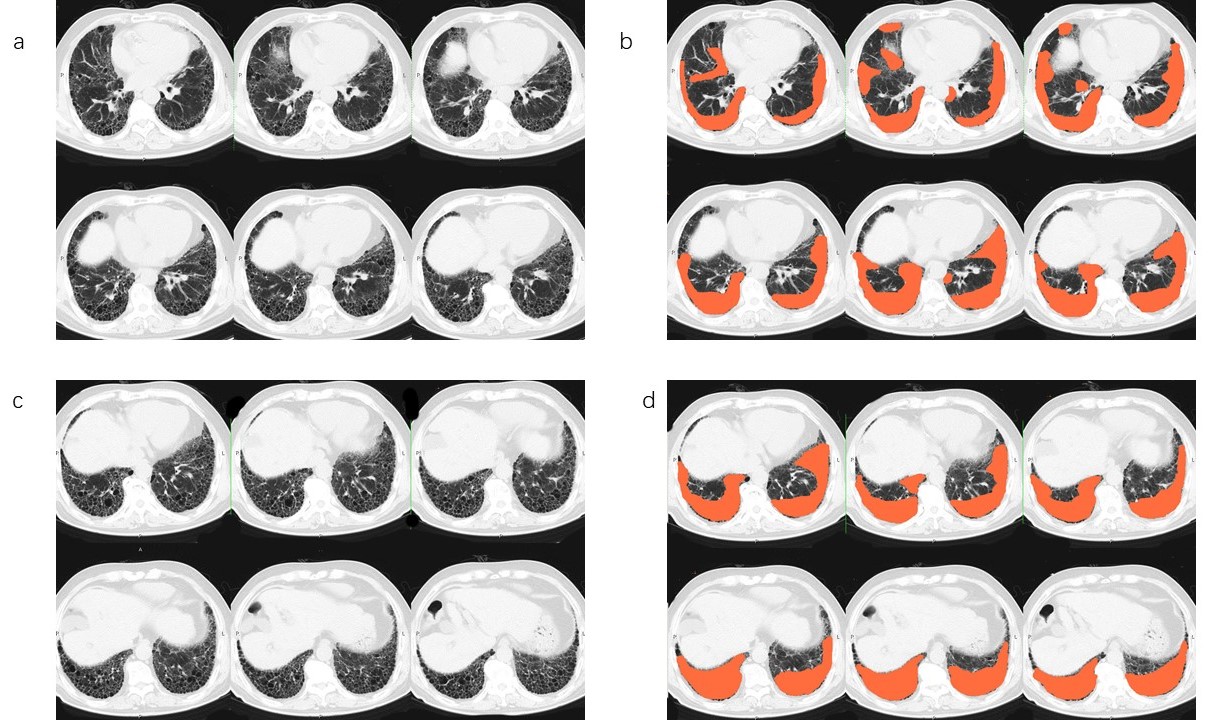

Supplement: Supplementary file 1 [file DataSheet4.DOCX]

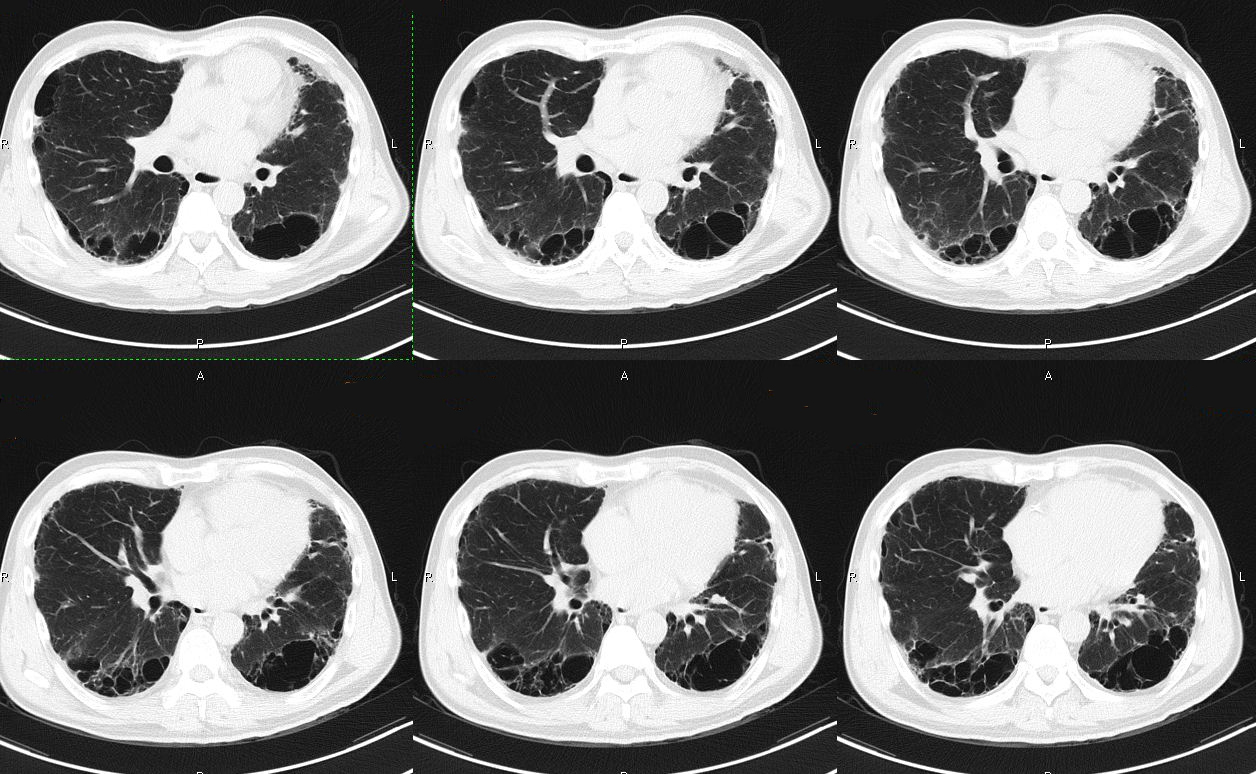


. **Figure S5a**. Original HRCT image


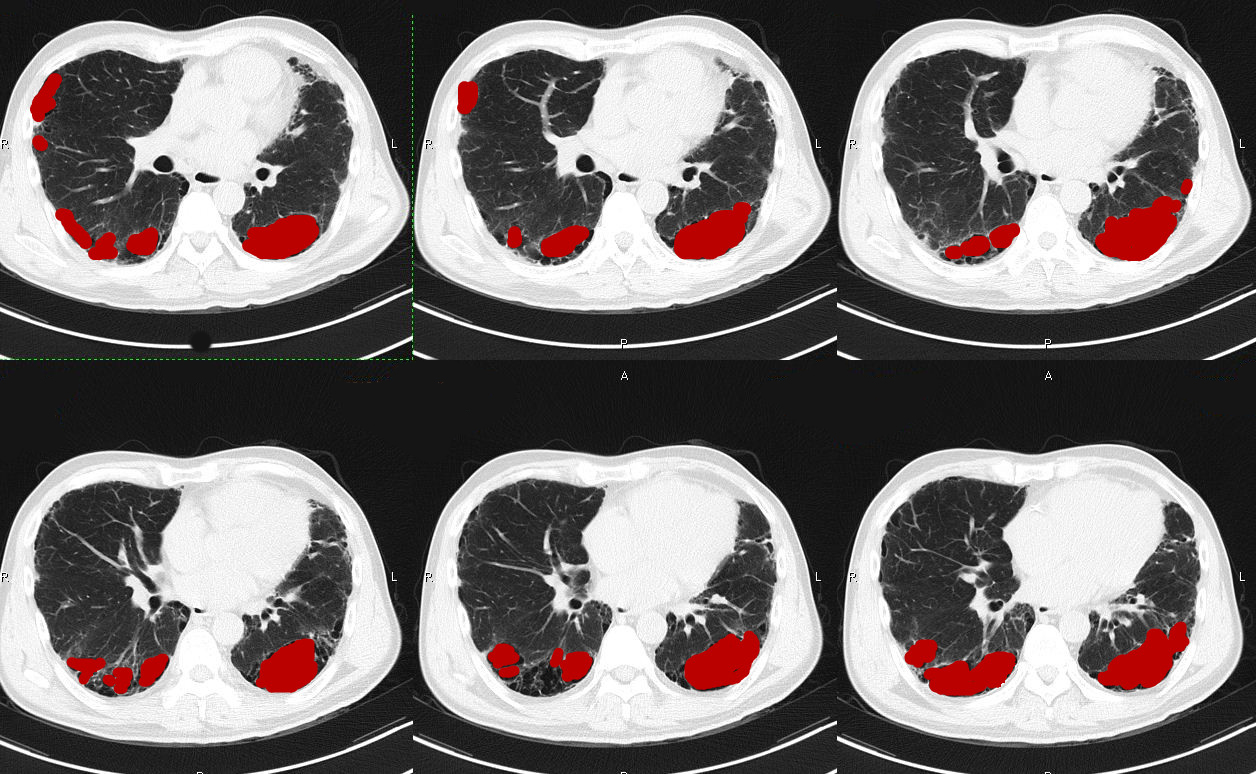


**Figure S5b**. HRCT image marked manually

Supplement: Supplementary file 3 [file DataSheet6.DOCX]

**Figure.S2** Honeycomb Lung Segmentation Process with Deep Learning


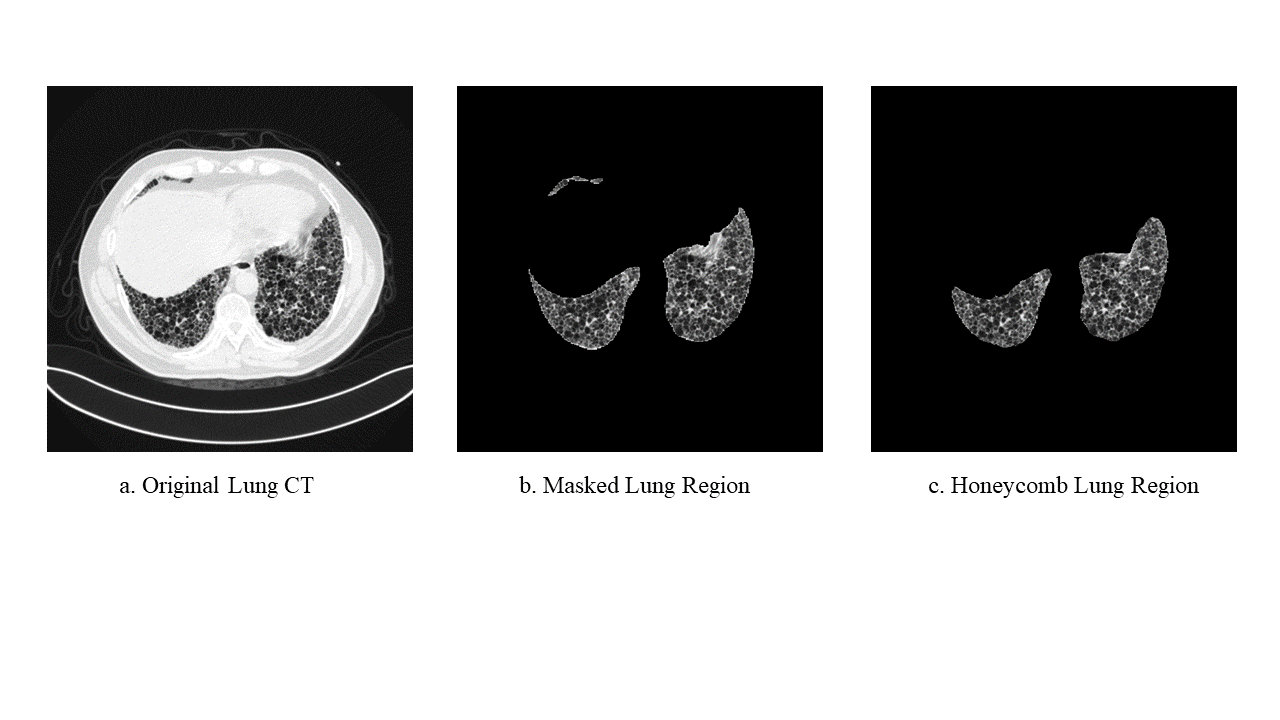

Supplement: Supplementary file 5 [file DataSheet3.DOCX]

**Figure S4.** Network architecture of FSN
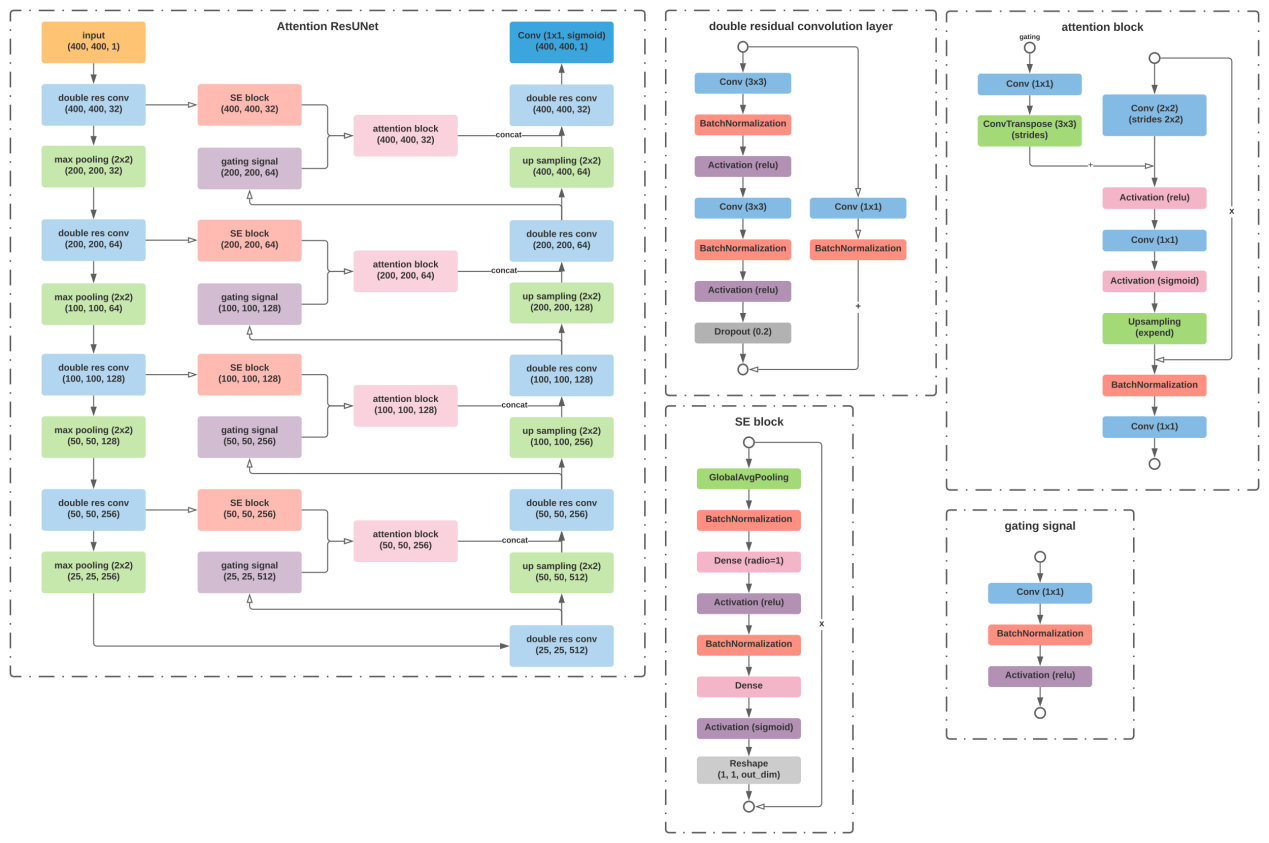

Supplement: Supplementary file 6 [file DataSheet5.DOCX]

**Figure.S1**: The flow chart of pulmonary fibrosis staging and severity assessment


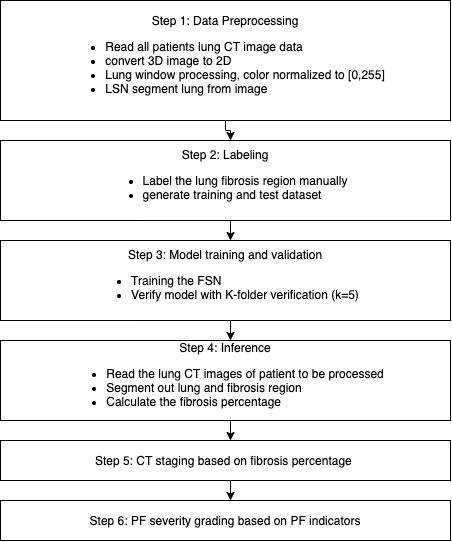

Supplement: Supplementary file 7 [file DataSheet2.DOCX]

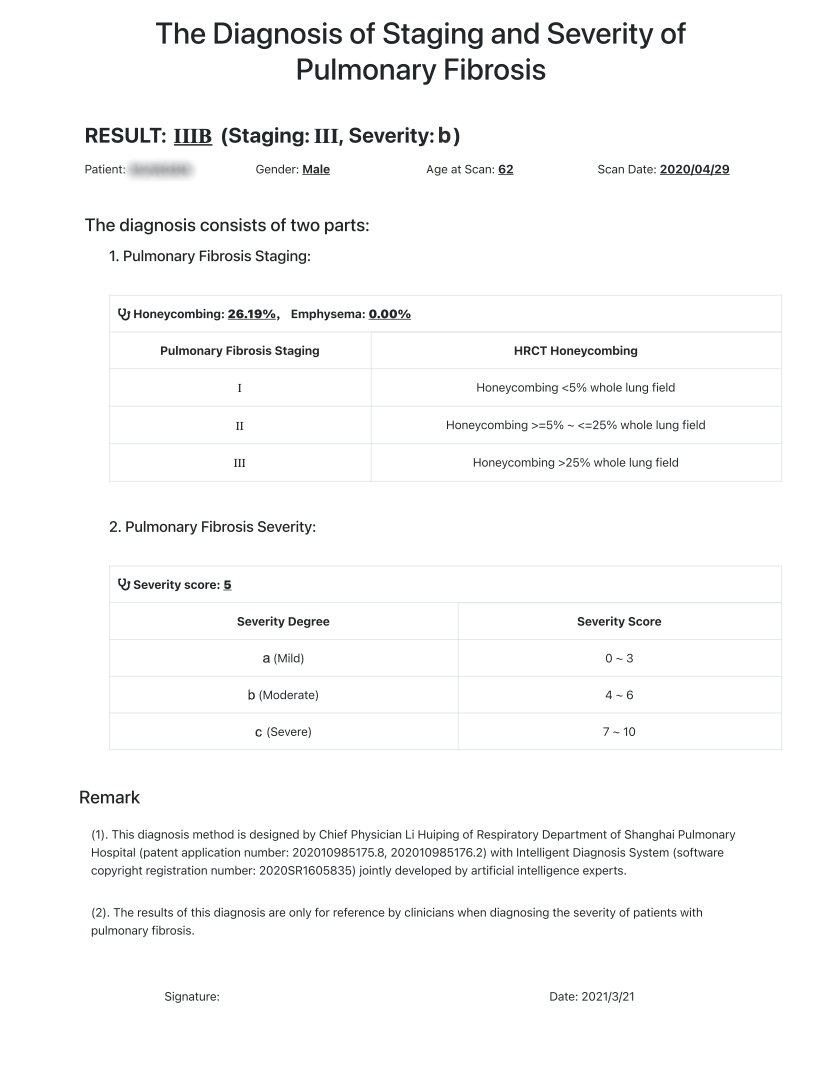
**Figure S7**. Evaluation Report

Supplement: Supplementary file 8 [file DataSheet8.DOCX]
