# Supplementary material for: Idiopathic Pulmonary Fibrosis Mortality Risk Prediction Based on Artificial Intelligence: The CTPF Model
Source: Front Pharmacol. 2022 Apr 26;13:878764. doi: 10.3389/fphar.2022.878764 (PMC9086624; doi:10.3389/fphar.2022.878764)
Supplement: Supplementary file 2 [file DataSheet7.DOCX]

**Figure S6.** Lung Bullae Segmentation Process with Deep Learning


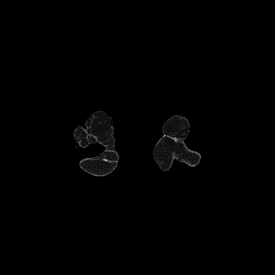

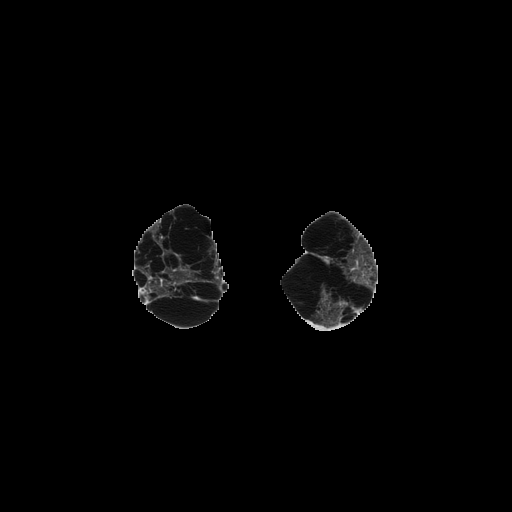

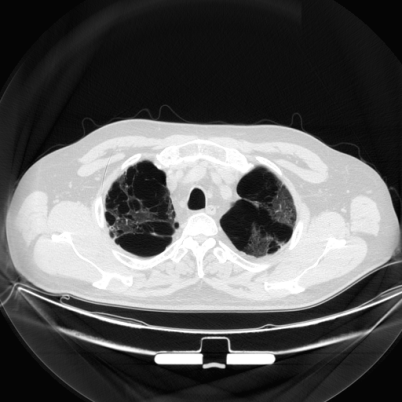


a. original HRCT b. Semantic segmentation of lung c. Semantic segmentation of Pulmonary
