# Supplementary material for: Idiopathic Pulmonary Fibrosis Mortality Risk Prediction Based on Artificial Intelligence: The CTPF Model
Source: Front Pharmacol. 2022 Apr 26;13:878764. doi: 10.3389/fphar.2022.878764 (PMC9086624; doi:10.3389/fphar.2022.878764)
Supplement: Supplementary file 4 [file Table1.DOCX]

**Table S1. K-fold cross validation**

| K* | Training overlap | Verify the degree of overlap | Test for fit |
| --- | --- | --- | --- |
| 1 | 0.8089 | 0.8013 | 0.7735 |
| 2 | 0.8672 | 0.8065 | 0.7713 |
| 3 | 0.8049 | 0.7743 | 0.7634 |
| 4 | 0.8121 | 0.8023 | 0.7802 |
| 5 | 0.8476 | 0.8043 | 0.7745 |
| Mean | 0.8281 | 0.7977 | 0.7726 |
| SD | 0.0248 | 0.0119 | 0.0055 |

* K-Folder Number of times of cross-validation
