# Supplementary material for: Idiopathic Pulmonary Fibrosis Mortality Risk Prediction Based on Artificial Intelligence: The CTPF Model
Source: Front Pharmacol. 2022 Apr 26;13:878764. doi: 10.3389/fphar.2022.878764 (PMC9086624; doi:10.3389/fphar.2022.878764)
Supplement: Supplementary file 9 [file DataSheet1.DOCX]

Supplemental material

**Supplementary Material 1. Establishing the deep learning model for pulmonary fibrosis semantic segmentation**

The process of pulmonary fibrosis segmentation of chest HRCT images was conducted as follows: **Figure S1.**

**Step 1 - Data preprocessing**: DICOM 3D chest CT image data was read for all 102 cases of IPF who were diagnosed from January 1st, 2005 to December 31st, 2010 in the Department of Respiratory Medicine, Shanghai Pulmonary Hospital patients, the 3D format then sliced into 2D images, lung window processing performed, and pixel values normalized to range 0-255. For each patient, 12 images with lesions were selected, totaling 1224 images, then a pre-trained lung segmentation network (LSN) was used to extract the lung region from the image and crop it to a uniform size of 400×400. LSN's function is to segment the lung region out of the 2D image to avoid the possible interference of the background image in the later step of fibrosis segmentation. The input to LSN is a slice of lung CT, shown in **Figure.S2a**; the output is a semantically segmented image of the lung region, shown in **Figure.S2b**.

**Step 2 -** **Manual labeling**: Based on the segmented lung output by step 1, we used software tools to label 1224 2D images manually with different colors representing the fibrosis lesion area **(Figure S3.**). Training and validation data sets were generated randomly according to a ratio of 8:2.

**Step 3 – FSN model training and verification:**

The fibrotic lesion segmentation network (FSN) was used to segment lung fibrosis regions based on the output of the LSN (**Figure S2b.)** in step 2, the output of FSN is shown in **Figure S2c.** The images were augmented (flipped and zoomed horizontally) during the training process, the number of training images was quadrupled from 980 to 3920, and the ratio of the number of images of training set images compared to validation set images was 16:1.

K-Folder Cross Validation (K=5) was used to validate the FSN model. The result is discussed below.

**Step 4- Inferencing**: The new patients’ CT images were fed into the image preprocessing pipeline, then processed sequentially by LSN and the FSN to segment the lung and fibrotic lesion regions in all CT images. Then we calculated the area of fibrotic lesion region and its percentage of the entire lung.

**Step 5 - Fibrosis staging**: Pulmonary fibrosis stage of the patients was classified according to the area percentage of the fibrotic lesion calculated in Step 4 according to two cut-off points.

**Step 6 - Severity grading**: The severity of the patient's pulmonary fibrosis was classified based on PF parameters.

**The performance of Fibrosis Segmentation Network**

The fundamental component of the fibrosis staging was performing the semantic segmentation of the fibrotic lesion region of lung CT **(Figure S2 a-c.**). The verification was carried out on the data set including 224 images of 20 patients with the K-Folder verification (K=5). The average DICE coefficient (2|X∩Y|)/(|X|+|Y|) was 77.26%, which was 8.39% higher than the benchmark (U-Net with the spatial pyramid pooling module) 68.78%, the standard deviation was 0.55% **(Table.S1).**

**Supplemental material 2.** **Network architecture of FSN (Figure S4.)**

Semantic segmentation of pulmonary fibrosis lesions CT image is a fundamental step. The semantic segmentation network (FSN) integrated the attention mechanism, SE-ResNet and Encoder-Decoder, and other neural network structures to achieve high accuracy in the case where only a limited number of noisy samples is available.

Model parameters:

- The deep neural network model has 61 layers.
- Total number of parameters is 9.95 million.
- The dimension of CT image input is (400,400,1).
- The dimension of image segmentation output is (400, 400, 1).

The deep learning network adopted the encoder-decoder architecture. The encoder and decoder aim to perform feature extraction and image restoration, respectively.

• Encoder: The purpose of the encoder is to extract features of pulmonary fibrosis, SE-ResNet takes both spatial and channel modeling capabilities into account.

1. SE module: Channel wise attention models the interdependency between channels, which enables the model to increase the sensitivity to the informative features.
2. Double residual convolution module: In addition to the conventional convolution module, the number of channels is controlled by 1X1 convolution.
3. Attention & gate signal module: The gate signal module uses lower-level features to guide attention to the lesion area.

• Decoder (the rightmost column):

1. The features are restored to the high-resolution image to ensure accurate segmentation, continuously connecting the output of up sampling and attention.
2. Provide input for the attention module.

**Supplemental material 3. Building semantic segmentation model of** **lung bullae and calculating its area percentage of the total lung (Figure S5 a-b; Figure S6 a-c)**

Pulmonary bullae are sac-containing cavities formed in lung tissue with a diameter greater than 1 cm. From an algorithmic perspective, the semantic segmentation of lung bullae is similar to pulmonary fibrosis; the only difference is different models trained by different data (including marking). Therefore, we reuse the same framework and network architecture of pulmonary fibrosis to build another semantic segmentation model for pulmonary bulla assessment. With the data of pulmonary bullae, we trained a new model for semantic segmentation of pulmonary bullae and calculated its percentage of the total lung area. The process as below:

- Selected CT images of 68 pulmonary bullae patients.
- Carried out data preprocessing steps.
- Segmented lung region with the same LSN.
- Marked pulmonary bullae regions manually with a total of 2160 layers (each patient with 30 levels on average), based on which training and test data sets were generated randomly with a ratio of 8:2.
- Trained the semantic segmentation models and perform verification with k-folder validation (k=5). The model’s DICE coefficient on the validation dataset is 72.84%.

Appendix: System environment

AWS EC2 GPU Instance: g4dn.2xlarge

vCPUs: 8 Intel Xeon P-8259L (2.5 GHz)

Memory: 32G

Storage: 1 x 225 (SSD)

GPU: Nvidia GTX 2080 (8G)

OS Environment:

Ubuntu 18.04 LTS

Python 3.7.7

Tornado = 6.0.3

Numpy = 1.18.1

Pandas = 1.0.3

SimpleITK = 1.2.0

opencv-python = 4.2.0

tensorflow-gpu = 2.2.0

torch = 1.5.0
